# caOmicsV: an R package for visualizing multidimensional cancer genomic data

**DOI:** 10.1186/s12859-016-0989-6

**Published:** 2016-03-22

**Authors:** Hongen Zhang, Paul S. Meltzer, Sean R. Davis

**Affiliations:** Genetics Branch, Center for Cancer Research, National Cancer Institute, National Institutes of Health, Building 37, Room 6138, 37 Convent Drive, Bethesda, MD 20892-4265 USA

**Keywords:** Software, R package, Genomic data visualization, Multidimensional data visualization

## Abstract

**Background:**

Translational genomics research in cancers, e.g., International Cancer Genome Consortium (ICGC) and The Cancer Genome Atlas (TCGA), has generated large multidimensional datasets from high-throughput technologies. Data analysis at multidimensional level will greatly benefit clinical applications of genomic information in diagnosis, prognosis and therapeutics of cancers. To help, tools to effectively visualize integrated multidimensional data are important for understanding and describing the relationship between genomic variations and cancers.

**Results:**

We implemented the R package, caOmicsV, to provide methods under R environment to visualize multidimensional cancer genomic data in two layouts: matrix layout and combined biological network and circular layout. Both layouts support to display sample information, gene expression (e.g., RNA and miRNA), DNA methylation, DNA copy number variations, and summarized data. A set of supplemental functions are included in the caOmicsV package to help users in generation of plot data sets from multiple genomic datasets with given gene names and sample names. Default plot methods for both layouts for easy use are also implemented.

**Conclusion:**

caOmicsV package provides an easy and flexible way to visualize integrated multidimensional cancer genomic data under R environment.

## Background

Featured with high-throughput technologies, current translational genomic research of cancers often generates multidimensional data such as mRNA/miRNA expression, DNA methylation, exome sequencing, and SNP/DNA copy number variations [1, 2]. Data analysis at multidimensional level will greatly benefit clinical applications of genomic information in diagnosis, prognosis and therapeutics of cancers. To help, tools to effectively visualize integrated multidimensional data are important for understanding and describing the relationship between genomic variation and cancers [3–5].

Visualizing multidimensional genomic data have been implemented in different ways: genomic coordinate based presentation, heatmaps, and networks views [4]. Genomic coordinate based tools such as UCSC genome browser and Integrative Genomics Viewer are powerful in viewing of detailed sequence and various types of variations as well as epigenomic and transcriptomes profiles that tied to genomic loci [6, 7], and CIRCOS and its implementation under different environments [8–10] help in exploring relationships between genomic alterations or positions. One disadvantage of genomic coordinate based tools is the limitations on numbers of samples and genes displayed simultaneous and integration of genomic variations with network/pathway information. In contrast, heatmap and network views can integrate multiple types of genomic variations independent of genomic loci in multiple sample groups at gene set or pathway level and are commonly used in presenting relationship between genomics variations and sample features and relationship between different genomic alterations [11–13].

The R statistical programming environment, an important open source tool used in cancer research community for statistical analysis and visualization of cancer genomic data, has packages which implemented genomic coordinate based views [14–16] and complex heatmap views [17]. To facility the R with more flexible and easy way in presenting multidimensional genomic information, we developed the caOmicsV package for R, to provide a set of graphic functions for visualizing multidimensional genomic data with two different types of layout: matrix layout (bioMatrix) and circular layout on biological network (bioNetCircos).

## Implementation

The caOmicsV package is implemented with R language only and provides two layouts for displaying multidimensional genomic dataset: bioMatrix and bioNetCircos layout. Both layouts support to display sample features, mRNA and miRNA expression, DNA copy number variations (CNV), DNA methylation data, and summarization data. On bioMatrix layout, clinical features of cancer samples are shown with different colored rectangles, gene expression (mRNA and miRNA) data are plotted as heatmap, DNA methylation status are presented as colored rectangle outlines, and DNA copy number variations are displayed as colored points. Besides gene names and sample names, summarized data can also be presented on the layout as text or bars. On bioNetCircos layout, a biological network is built from given gene expression dataset and genes are presented as nodes on the network. Clinical features, mRNA and miRNA expression, DNA methylation, DNA copy number variation, and other summarized data for each sample are displayed in circular layout on each node (gene) as polygons, heatmap, bars, points, or lines. In the center of each node, link lines could be plotted to display the relationship between two samples. Both layouts are using low level plot functions of R graphics package. For bioNetCircos layout, installation of R igraph package is required.

## Results

**Fig. 1:**
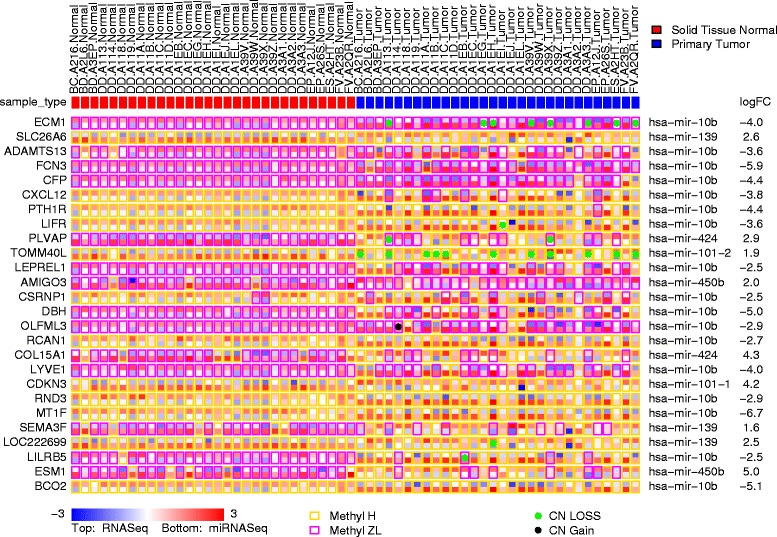
Output of default bioMatrix plot method. Sample information is plot on the top of matrix and below that are data for each gene. In each gene row, each column represents a sample, and mRNA and miRNA expression are shown as heatmap, DNA methylation is represented by different colored outlines, DNA CNV are plotted as colored points. For each gene, top half heatmap show mRNA expression and bottom half are expression of miRNA that is most significant negatively related to the gene. The mean fold change for each gene is listed at the most right of plot area

**Fig. 2:**
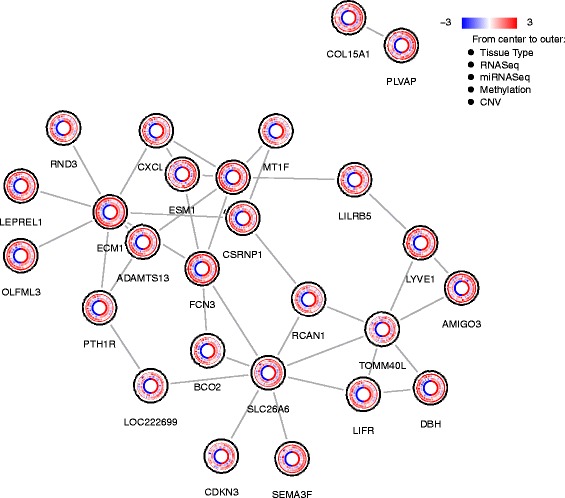
Output of default bioNetCircos plot method. The biologic network is built with igraph package and each node represents a gene. On each node, from most inner to outer, are sample groups (polygons), mRNA expression (heatmap), expression of miRNA that is most negatively related to the mRNA expression (heatmap), DNA methylation (bar), and DNA CNVs (points). All gene labels are put under the nodes by default

The presentation of multidimensional genomic information and sample feature with caOmicsV package is shown in Figs. 1 and 2. Two default plot methods, plotBioMatrix() and plotBioNetCircos() are implemented for easy use. As shown below, simply pass the input data to relevant plot functions will generate the images with default parameter setting as Figs. 1 and 2.

Default plot method for bioMatrix plot

- library(caOmicsV)
- data(biomatrixPlotDemoData)
- plotBioMatrix(biomatrixPlotDemoData, summaryType = "text")
- bioMatrixLegend(heatmapNames = c("RNASeq", "miRNASeq"),
- categoryNames = c("Methyl H", "Methyl L"),
- binaryNames = c("CN LOSS", "CN Gain"),
- heatmapMin = −3, heatmapMax = 3, colorType = "BlueWhiteRed")

Default plot methods for bioNetCircos plot

- library(caOmicsV)
- data(bionetPlotDemoData)
- plotBioNetCircos(bionetPlotDemoData)
- dataNames < − c("Tissue Type", "RNASeq", "miRNASeq", "Methylation", "CNV")
- bioNetLegend(dataNames, heatmapMin = −3, heatmapMax = 3)

The input data format for both bioMatrix and bioNetCircos layout plot is a list of data matrix and character vectors. A function getESet() was implemented in caOmicsV package to build the input data list, which take s given set of gene names, sample names, and plot data in data frame format as input and returns a list containing all plot data. To help in preparing input dataset for getESet() function, a set of supporting functions are also provided in the package including of methods of extracting subset data from big data set with given gene names and sample names as well as sorting datasets for desired orders required for the plot methods.

**Fig. 3:**
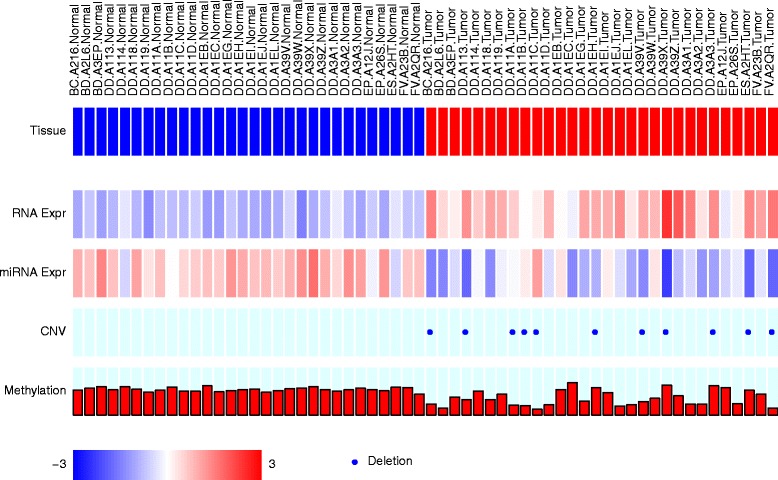
Demo of customized bioMatrix layout plot to display sample information with heatmap, points plot, and bar plot for only one gene

Beside of the default plot methods, caOmicsV package can also allow users to generate customized images with each specific plot function. Figure 3 is a demo of customized bioMetrix plot which displays sample information, mRNA expression, miRNA expression, DNA CNV, and methylation for one gene. Also, caOmicsV plot are implemented with low level plot methods of R graphic package, more decoration items such as title and extra legend could be easily added onto the plot outputs. With the R graphic layout supporting, multiple caOmicsV plots could be generated on one image. In addition, caOmicsV plot functions use data matrix as input format, other data other than genomic data, when correctly formatted, may also be plotted with caOmicsV package.

## Conclusions

caOmicsV package provides sample way to present integrated multidimensional genomic data under R environment with both matrix layout and circus layout on biological network.

## Availability and requirements

**Project name**: caOmicsV

**Project home page:**https://www.bioconductor.org/packages/caOmicsV

**Operating systems**: any operating system supporting R

**Programming language**: R

**Other requirements**: working R installation

**Licence**: GPL

**Any restriction to use by non-academics**: none
